# Auditory Middle Latency Evoked Responses: A Standardizing Study

**DOI:** 10.1016/S1808-8694(15)30060-4

**Published:** 2015-10-19

**Authors:** Francisco Sales de Almeida, Paulo Roberto Pialarissi, Luiz Eduardo Ferreira Paiva Júnior, Maria Aparecida Oliveira Almeida, André Silva

**Affiliations:** a PhD at USP/SP, member of the medical staff at the Odontomed Hospital.; b Doutor em Otorrinolaringologia pela USP, Professor Titular da Pontifícia Universidade Católica de São Paulo.; c Graduando em Medicina, Acadêmico da Faculdade de Medicina de Itajubá.; d Pós-graduanda em Audiologia pelo CEDIAU, Fonoaudióloga do Hospital Odontomed.; e Graduando em Medicina, Acadêmico da Faculdade de Medicina de Itajubá. Hospital Odontomed.

**Keywords:** Auditory middle latency response, Auditory potentials, Auditory electrophysiology

## Abstract

The presence of auditory middle latency evoked responses allows us to make an evaluation of the peripheral and central auditory system integrity, as well as the nucleus and auditory ways existing until the level of the thalamus-cortical region and primary auditory cortex. **Aim:** Our objective is to evaluate the presence or not of this numerous peaks, as well as, their latencies and intervals and extend to make a standardizing study. **Way of study:** Contemporary study of Coorte with transversal cut and the outline was quantitative, descriptive e non experimental. **Material and method:** Studying several peaks, positives and negatives, caused by the middle latency auditory potentials, in a population of young adults individuals, ranging from 18 to 30 years old, from both genders, with normal hearing. It was used a monoauricular resonant stimulation and a capitation, separately, in both cerebral hemispheres, with surface electrodes. **Result:** In this research was verified that the analyzed crossings did not present statistically significant results and it was stipulated a pattern from the obtained results. Based on the non-statistical difference found we can affirm that to the Po waves was obtained respectively an average and standard deviation of 12,09 and 1,84; for Na 17,91 and 2,60; for Pa 29,41 and 5,66; for Nb 41,43 and 8,89; to for 51,44 ms e 12,63 and finally to the interval Na-Pa 11,52 and 4,99. **Conclusion:** 1- The presence of the defluxions Po, Na, and Pa was verified in all the registers, whereas the defluxions Nb and Pb were absent in only 06 registers. 2- By having these registers, we evaluated that the inveiglement of these defluxions can be used as a reliable method to detect the average latency of the auditory potentials, by electrical generated activities, possibly in sites located in the thalamus-cortical area, evoked by sonorous stimulation. From that point, we establish a pattern of responses for young ones with normal hearing, helping us with future studies in patients with alterations in the hearing system.

## INTRODUCTION

It is highly important to capture auditory middle latency evoked potentials (AMLR) in trying to objectively enhance auditory function assessment in patients with hearing loss, both to determine auditory limits and to study the latency period and values of the amplitudes related to its various deflections.

These potentials represent a series of deflections, positive and negative, which occur between 10 and 18 ms after the beginning of the auditory stimulus, which are located after brain stem evoked potentials, preceding late responses, related to cortical and cognitive functions.

These responses were registered for the first time at the “Massachusetts Institute of Technology” by Geisler et al.^1^, in 1958, using at the time, a computerized device for measuring responses. These authors infer that the waves observed in their work were representative of auditory afferent activities related anterior regions in the brain cortex.

Nevertheless, in 1963, these responses were considered generators purely of myogenic potentials^2^. For this reason, these potentials were no longer being deemed valuable for the evaluation of afferent auditory pathways.

From 1967 on there have been studies demonstrating the clinical applicability of these responses^3^, which has been supported by registries with surface electrodes obtained from patients who underwent brain surgeries.

In 1974, various waves were described, elicited by sound stimuli, among which, N18 (Na), P30 (Pa) and P50 (Pb), already trying to distinguish and separate them from the purely myogenic responses, such as the ones that occur by contraction of the post auricular and temporal muscles^5^.

Later studies^6^ have shown the presence of these waves even under muscle paralysis obtained through the administration of succinylcholine. These observations were reinforced by other following studies in which an anesthetic agent (Fentanyl) was used associated to pancuronion which led to muscular anesthesia^7^.

Various authors, from then on, have done studies related to the latency wave generating sites between 10 and 80 ms, both in animals, differentiating the contributions of the primary sensorial portions from those originated in the extraleminiscal pathways of the auditory system, for example, reticular substance, suggesting the importance of the thalamus cortical^8^ pathways, and in patients subject to neurosurgeries, using surface electrodes, describing in detail the various waves and their starting points^9^.

Despite of such evidences, the more systematized studies on these responses used to evaluate the afferent auditory pathways only started to gain importance at the late 80’s.

In our field of study, we may mention one on the auditory electrical potentials of middle latency in normal female individuals, with the objective of determining latency times for positive deflections (Pa, Pb e Pc) and negative deflections (Na, Nb, Nc) in relation to the stimulated side and the frequency in which each of these deflections are seen^10^, and, more recently, another study detecting these responses in children and teenagers with normal hearing^11^.

Various authors consider that the capture of auditory middle latency potentials have great clinical applicability in various situations, such as the electrophysiological determination of the auditory limits in the low frequency range, in evaluating the functioning of cochlear implants, to evaluate how the auditory pathways are functioning, the location of lesions in the auditory pathways and some intraoperative applications^12^.

These evoked potentials may be utilized for the evaluation of information processing, as we have seen studies, when analyzing concomitantly the auditory middle latency potentials in the somatosensorial and auditory modalities, more specifically P50, in healthy male individuals. This study supports the theory of deficiency in the processing of information in squizofrenic individuals (defect in P50)^13^.

Trying to explore other aspects of information processing, the habituation of evoked auditory responses was studied (P50) utilizing repetitive stimuli. We observed, then, that the amplitude of the P50 response to the second of two homologous stimuli was significantly less reduced in patients with migraine then in healthy volunteers^14^.

It was suggested that the ascending reticular activity seems to be affected in some patients when studying the auditory middle latency evoked potentials in patients with obstructive sleep apnea syndrome, before and after treatment. After the apnea treatment, there was a significant improvement in the nocturnal hypoxia and also an increase in middle latency P1 peak width and these potentials had a better distribution on its electric field on the scalp^15^.

The capture of auditory responses from the brain stem and the auditory responses of middle latency have also been used to study a group of patients with tinnitus, in comparison with registries obtained from normal and from older individuals. In the tinnitus and elderly individuals groups, when looking at brain stem potentials, changes in relation to the VII wave were observed, and in regards of the middle latency potentials, many wide waves occurring in some of the components of this group were observed, but not in all of them. There has been, also, the widening of these middle waves without the corresponding widening of brain stem potentials. This suggests, in the authors’ opinion, that there could be a selective middle latency wave generator in patients with tinnitus and distinct effects of age over cochlear physiology^16^.

Comparative analysis, when capturing auditory middle latency evoked potentials through electroencephalic registers and through magnetoencephalic data suggest multiple supra temporal sources for the various deflection s observed. Are correlated, then, Pa (28ms) to the medial portion of Heschl’s gyrus; Nb (40 ms)/Pb1 (52 ms) to the lateral face of the supratemporal gyrus; and Pb2 (74 ms) to the anterolateral portion of the Heschl’s gyrus^17^. These findings are in accordance with previously invasive intracerebral registries and with studies in animals, which describe the secondary areas involved in the generation of auditory middle latency evoked components.

The capture of these responses may be impacted by external agents, such as alcoholic beverages^18^ or anesthetic drugs^19^.

We may register them to study patients with hearing disability^20^.

Also studying the auditory short, middle and long latency evoked potentials, it was verified the reliance of the registry of these potentials, when doing the test-retest procedure, in a one year time frame, both in young adults and in elderly individuals (between 21 and 92 years old)^21^.

When doing the simultaneous capture of intracerebral auditory evoked potentials directly from the auditory cortex and the median geniculated body^22^ in a patient, it was seen an initial negative response generated at the level of the median geniculated body, with latency around 13.5 ms, and two positive peaks (P21 and P29), with wider ranges for low frequency sounds suggesting, then, the existence of a possible tonotopic organization of this nucleus. It is observed, also, that peaks originated in the thalamus activity were strongly overlapped with the cortical activity registered on the Heschl’s gyrus before the 30 ms period (with N13 preceding the first cortical component in 3.5 ms; whereas P21 and P29 precede and follow, respectively, in relation to the two cortical responses that follow, which show reverse polarity, in a 1.5 - 2 ms interval). This study shows new functional data over the activity of the median geniculated body and suggests a more complex role of the thalamus in sound perception.

One may also infer that the capture of these potentials is extremely useful to evaluate patients with cochlear implants^2^, since these can be produced through electric waves, having the advantage over brain stem potentials, because its latency periods are bigger and are not mistaken for the generating stimuli.

These potentials represent an important tool to evaluation brain function, not only from an auditory perspective, but also in patients with neurological involvement, as has been demonstrated in various studies with comatose patients or patients who suffered head trauma^24^.

In the last years many scientific studies have been done, showing that these potentials are related to the nuclei and the auditory pathways situated in the thalamus cortical region and the primary auditory cortex, mainly the thalamus-cortical^25^, ^26^, ^27^.

Nevertheless, in our country, little has been produced on this matter.

We still lack a higher standardization for our population, as to the appearance of various positive (Po, Pa and Pb) and negative (Na, Nb) deflections, according to their order of appearance, its respective latencies and amplitudes and on what relates to the stimulation of only one ear or both, concomitantly. Taking these data as our basis, we can create a test protocol adapted to our reality, with the goal of studying the variables which would interfere in the capture of the middle latency potentials, using auditory stimuli, either with a click or with tone burst.

Based on these evidences, we propose to study a group of normal patients and evaluate the presence of various deflections corresponding to the auditory middle latency potentials, as well as its latencies and amplitudes, with the following objectives:

1 - Identify the positive and negative deflections on the traces of the auditory middle latency evoked responses (situated between 10 ms and 80 ms after the sound stimuli) in a population of young adults, with normal hearing.
2 - Analyze the characteristics of these responses (latency of each deflection and Na-Pa interval) and do its standardization for the population studied.

## MATERIALS AND METHODS

The alignment was quantitative, descriptive, and non-experimental. It was a contemporary cross-sectional cohort study. The data were collected in the laboratory. The study was a comparison among individuals.

The sample was a group of 50 individuals, young adults of both genders, being 34 male and 16 female subjects, living in Itajubá. The subject of the study was the individual. The sampling was a non-probabilistic one and by convenience. The exams took place in Odontomed Itajubá Hospital, in the department of otorhinolaryngology.

As inclusion criteria, young adults were studied, with ages ranging between 18 and 30 years old, with normal hearing and without previous history of otological and/or neurological diseases.

The history of the participating individuals was obtained, and they went through clinical otological inspection, acoustic immittance measurements, stapedian reflex study, conventional tonal audiometry (frequencies tested between 250 Hz and 8000 Hz) and logoaudiometry to assure auditory health.

The capture of middle latency potentials was done with both the equipment and the individuals situated inside an acoustic sound proof booth, to avoid electrical interferences of any nature (for example, static electricity). With this, there would only remain the proper interferences of the individuals being tested or the examiner.

We tried to avoid these specific interferences through additional care measures. The electrodes, as well as the earphones, were positioned by the examiner, after instructing the individual. The environmental conditions in the booth (temperature, light, silence, positioning) were the most adequate to maintain the individual comfortable and relaxed, without, though, allowing the person to fall asleep. The electrodes were placed, after careful skin cleansing, with adequate electrolytic paste, allowing for a better capturing of the potentials being studied. The exam was only started when the base line of the electroencephalogram was stable, without interferences.

The equipment used is the operational system CE - EP 25. The label CE indicates that “Interacoustics AS” meets the requirements of the VI annex of medical directions 93/42/EEC and the equipment was approved by TÜV, with identification nº 0123.

This equipment, additionally, consists of:

• Electrodes: one as live, two as references and another as ground (indifferent) allowing for the individual analysis of each brain hemisphere.
• Pre-amplifier: EPA 25, with two channels, obtaining a gain of up to 80 dB, which works within a range of frequency up to 8,000 Hz.
• Impedance measurer: with information regarding each electrode individually.
• Sound stimulator: the stimuli might be presented ranging from 1.1 to 80.1/sec, through earphones 3A ABR with insertion modes, calibrated in an IEC 126 coupler or earphones TDH 39. It produces “clicks” of 100 mms in three polarities (condensation, rarefaction, alternate) and tone burst in the frequencies 500; 1,000; 2,000; 3,000 and 4,000 Hz. Level of stimulation between 20 and 130 dB. Sound pressure level- “SPL” (-10 to 100 dB Hearing Level “HL”). White masking noise, calibrated in SPL, with a masking levels from 0 to 40 dB relative to the stimulus.
• Recording: up to 900 ms, with a gain of 74 to 104 dB (automatic or manual selection). Has low pass filters (none or ranging from 17 Hz up to 12,000 Hz), high pass filters (none or ranging from 0.83 Hz up to 500 Hz) and analogue filters (from 0.5 Hz).
• Operational system: Windows 98.
  The exam protocol used in our research, to capture middle latency potentials, was as follows:
• Stimulus: “click”, of 100 ms duration, with alternate polarity, without the use of masking, in a frequency of 7 stimuli per second, up to a total of 1,000 stimuli.
• Intensity of the stimulus: 70 dB SPL, presented through ear phones TDH 39.
• Electrodes: the ground electrode placed on the glabellum, the live electrode placed on the superior portion of the scalp, halfway between the cranial top and the mastoid region, over each cerebral hemisphere, and two “reference” electrodes, each placed over each ear lobe.
• Impedance: electrode impedance around 2W, being acceptable up to a maximum of 3W.
• Registration period: from 0 to 80 ms.
• Filters: 10 Hz high pass filters and 1,200 Hz low pass filters were used.
• Reproducibility index: 95%
• Mono-aural stimuli
• Sequence of stages of the stimuli and capture of answers:
  Stimulation Capture (*placement of the live electrode)
  1. Right Ear Right Brain Hemisphere (RERH)
  2. Left Ear Right Brain Hemisphere (LERH)
  3. Right Ear Left Brain Hemisphere (RELH)
  4. Left Ear Left Brain Hemisphere (LELH)

This way, we made 4 isolated registries in each of the 50 individuals, to a total of 200 registries of auditory middle latency evoked potentials.

Results obtained in each of the stages mentioned above were plotted separately, being compared to the results obtained from each of the stages.

Study parameters of the auditory middle latency responses (latency and intervals) were processed electronically, using MS Excel.

We considered, for analysis, the positive and negative deflections which were shown within the time frame from 10 ms to 80 ms after the sound stimulus. The positive deflections were named Po, Pa and Pb, while the negatives were named Na and Nb, according to the order of appearance. The latencies and the intervals were measured in milliseconds (ms).

The results obtained were subject to descriptive statistical analysis consisting of independent variables: percentages, frequencies, standard deviation, average and number of validated cases. The “Student t test” was used with acceptance of the significance level below 0.05.

We established variables overlapping: Po, Na, Pa, Nb, Pb waves and Na-Pa interval latencies, respectively on the sequences RERH, LERH, RELH and LELH, to establish its significance level

The relevance of the study is to establish a normality standard for different variables (latencies and intervals) in relation to various registered waves.

All the participants were explained the procedures to which they would be submitted and research goals, having accepted to participate in the study.

This study consisted of non-invasive evaluations which did not lead to any damage to the patient.

The research, which involved the participation of human beings, followed strictly the ethical rules established according to resolution no 196/96, of October 10, 1996 by the National Health Council, of the Health Ministry.

This study was approved by the ethics committee in research of the Wenceslau Braz Nursing School of Itajubá.

## RESULTS

The registry of the variables (latency of each Na-Pa interval) were obtained almost totally from the individuals examined, and can be seen on table 1.

**Table 1:** Analysis of registries obtained in relation to the latency of each wave (Po, Na, Pa, Nb and Pb) and the Na-Pa interval, with minimum and maximum values, their averages and Standard deviations related to the individuals participating in the study.

Stimulus x Capture	Statistical analysis	Po Latency (ms)	Na Latency (ms)	Pa Latency (ms)	Nb Latency (ms)	Pb Latency (ms)	Na-Pa interval (ms)
	Valid numbers	50	50	50	49	49	50
	Minimum	8,33	12,67	19,33	21,67	3,33	3,33
RERH	Maximum	18,00	24,33	37,33	68,33	75,67	21,67
	Average	12,10	18,01	29,00	40,59	49,59	10,99
	Standard deviation	1,91	2,34	4,49	7,88	12,58	4,22
	Valid numbers	50	50	50	49	49	50
	Minimum	5,67	11,00	18,00	24,67	29,00	3,00
RELH	Maximum	20,00	27,00	47,67	62,67	84,67	29,00
	Average	11,97	18,21	29,71	42,11	52,09	11,48
	Standard deviation	2,12	3,09	6,26	9,16	12,66	5,36
	Valid numbers	50	50	50	48	48	50
	Minimum	5,67	13,67	17,00	21,33	25,00	2,33
LERH	Maximum	17,00	24,00	38,67	59,33	77,00	24,00
	Average	12,25	17,42	28,42	39,56	49,85	11,12
	Standard deviation	1,91	2,34	5,23	8,48	12,18	4,94
	Valid numbers	50	50	50	48	48	50
	Minimum	9,67	12,67	19,00	22,67	24,00	3,33
LELH	Maximum	15,67	25,00	47,67	67,67	84,33	26,67
	Average	12,05	18,02	30,51	43,43	54,25	12,49
	Standard deviation	1,41	2,56	6,40	9,70	12,89	5,38

**Obs. RERH:** right ear right cerebral hemisphere, **RELH:** right ear left cerebral hemisphere, **LERH:** left ear right cerebral hemisphere, **LELH:** left ear left cerebral hemisphere.

On this table we show the values corresponding to the averages of each deflection latency, positive and negative, and that of the interval Na-Pa. Also are shown the minimum and maximum values, and the Standard deviation for each of these variables. It also shows the number of examinations valid for this analysis.

**Graph 1:**
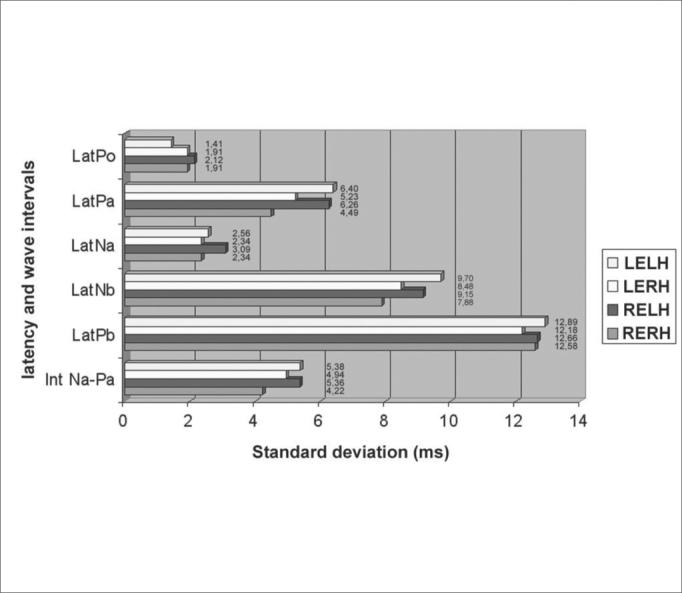
Analysis of the registries obtained for each wave (Po, Na, Pa, Nb and Pb) and the Na-Pa interval, with standard deviation values, in the various criteria of stimulation and capture, related to the individuals participating in this study.

The Standard deviation, for each of the variables, is presented on graph 1, representing the four forms of study, RERH, LERH, RELH and LELH.

Four tests were made with each Individual, adding to a total of 200 tests in a whole. As seen on table 2, the Po, Pa and Na deflections were registered in all tests, while the deflections Nb and Pb were absent in 6 tests.

**Table 2:** Analysis of the registries obtained in relation to the latency of each wave (Po, Na, Pa, Nb and Pb) and the Na-Pa interval, with minimum and maximum values, their averages and Standard deviations related to the individuals participating in the study and adding the valid registries pertinent to each wave.

Stimulus x Capture	Statistical analysis	Po Latency (ms)	Na Latency (ms)	Pa Latency (ms)	Nb Latency (ms)	Pb Latency (ms)	Na-Pa interval (ms)
	Valid numbers	200	200	200	194	194	200
	Minimum	5,67	11,00	17,00	21,33	3,33	2,33
All	Maximum	20,00	27,00	47,67	68,33	84,67	29,00
	Average	12,09	17,91	29,41	41,43	51,44	11,52
	Standard deviation	1,84	2,60	5,66	8,89	12,63	4,99

On table 3, we see the significance values in relation to response overlapping obtained from each stage of stimulation and capture.

**Table 3:** Analysis of the registries obtained in relation to the latency of each wave (Po, Na, Pa, Nb and Pb) and the Na-Pa interval of the various capture positions in individuals participating in this study through Student “t” test.

Stimulus x Capture	Po Latency	Na Latency	Pa Latency	Nb Latency	Pb Latency	Na-Pa interval
RERHxRELH	0,70	0,55	0,21	0,53	0,92	0,89
LERHxLELH	0,82	0,53	0,74	0,49	0,40	0,35
RERHxLERH	0,75	0,51	0,72	0,38	0,33	0,61
RELHxLELH	0,57	0,08	0,22	0,04	0,08	0,19
RERHxLELH	0,89	0,17	0,98	0,11	0,07	0,12
RELHxLERH	0,50	0,26	0,15	0,15	0,37	0,73

## DISCUSSION

We can detect the presence of various deflections, positive and negative, in a period from 10 ms to 80 ms after the sound stimulus, in the almost totality of the registries, within the parameters observed by other authors^11^, ^17^, ^22^. This makes the middle latency potentials capture examination an important tool used in the functional and topographic diagnostics of auditory lesions^12^, ^20^, as well as in examining information processing^13^, ^14^.

We have adopted, in our study, the nomenclature accepted by various authors considering the many deflections according to the latency period. Thus the positive waves were named Po, Pa and Pb, and the negative waves were named Na and Pa^5^, ^9^, ^10^.

The Po, Na and Pa deflections were present in all 200 registries, while the Nb and Pb deflections were absent from 6 registries.

Since no statistically significant difference was found, we may infer that the response found is similar in all the values studied. Based on this statement, all other values obtained from the captures, RERH, LERH, RELH and LELH, were added up establishing one only average and standard deviation value for each variable.

For the Po wave, we obtained, respectively, the average of the latency period and the standard deviation corresponding to the values of 12.09ms and 1.84; for the Na wave, 17.91 ms and 2.60; for the Pa 29.41 ms and 5.66; for Nb 41.43 ms and 8.89 and for Pb 51.44 ms and 12.63.

In relation to the Na-Pa interval, the average value was 11.52 ms and the standard deviation was 4.99.

Despite not having done a research study over points of generation for the various waves, we may infer that based on their latency periods, these deflections are generated in sites located in the thalamus cortical region and primary auditory cortex and/or primary cortical regions, as we have widely seen in the literature^8^, ^9^, ^17^, ^22^.

We believe, then, that these various waves are generated from sound stimulation in afferent auditory pathways anterior to the brain cortex, as initially observed by Geissler et al. in 1958^1^.

Based on these statements, we may consider the registries of the Po, Na and Pa deflections fundamental to verify the integrity of the auditory pathways, to a thalamus cortical and primary auditory cortex region level^12^, ^25^, ^26^, ^27^.

Thus, this study could be used still as a method of evaluation of patients with neurological problems (such as head trauma or coma)^4^, ^24^, as well as to follow up individuals with cochlear implants^12^, ^23^ or, even, in the topographic evaluation of patients with tinnitus^16^. These matters might be subject for later studies.

As referred to in the literature, these potentials tend to maintain the same registration standard through the test-retest research within a time frame of one year^21^, indicating a high level of confidence.

We have analyzed the registries obtained in relation to the location of the auditory potentials considering the positioning of the electrodes, over the specific region of each brain hemisphere. When these results obtained overlapped we could not notice any significant differences between one capture region and another.

We have observed the possibility of capture at the same time of both the auditory middle latency evoked responses (nuclei and auditory ways of the subcortical and primary cortical regions) and the auditory short latency evoked responses (nuclei and auditory ways of the brain stem). This makes the present this study a tool of extraordinary importance in daily clinical practice.

After making this standardization, as seen previously, the capture of these middle latency potentials presents us with a great possibility for further studies, under both the auditory and neurological perspectives. It allows us to electrophysiologically evaluate the auditory level of the individual, and gives us the possibility of a broader way of establishing the lesion area, in case of retrocochlear diseases.

The responses obtained after a stimulus on one ear and with ipsilateral capture, when overlapped with the responses obtained from the other ear under the same conditions, and also, when overlapped with the responses obtained counterlaterally, do not bear statistical significance.

The analysis of the amplitudes for each isolated wave or in a composition of two of them (for example: Na-Pa) will be made in another study, for verification of its significance and importance, through specificity and sensitivity.

We haven’t done research over these potentials under the use of drugs or on patients under anesthesia, although these situations might become topics for future studies^18^, ^19^.

The similarity of answers of the standard deviations presented in graph 1 reflects that there is little difference between data spread referring to the specific variables analyzed, and that this difference, as shown in table 3, has no statistical significance. The answers in graph 1 illustrate that the values of each of the variables are dispersed in a similar way for stimuli in either ear and in captures from any hemisphere in patients with normal hearing and with no previous history of any retrocochlear disorder.

From this data we may infer that a retrocochlear disease might alter these values in a significant way, making it possible, in the future, to create clinical and/or laboratory standards of specific middle latency potential. This type of exam will, in clinical practice, establish a disorder in the auditory anatomy and physiology, allowing for a sensitive or even specific exam.

## CONCLUSION

We may conclude that:

1. The presence of Po, Na and Pa deflections in all registries was shown, with an average of the latency period of 12.09 ms for Po, 17.91 ms for Na and 29.41 ms for Pa; while Nb and Pb deflections were absent from only 6 registries, and the averages of their latency periods were 41.43 ms and 51.44 ms respectively. The average of the Na-Pa interval was 11.52ms.
2. Through these registries, we conclude that the capture of these deflections give us a reliable method to detect auditory middle latency potentials, occurring from electric activity generated, possibly, in sites located in the thalamus-cortical region, evoked with the use of sound stimuli. Based on that, we established a response standard, among us, for young individuals, with normal hearing, allowing us to further study patients with alterations in the auditory system.
